# The impact of the COVID pandemic on working age adults with disability: Meta‐analysis of evidence from four national surveys

**DOI:** 10.1111/hsc.13882

**Published:** 2022-06-19

**Authors:** Eric Emerson, Zoe Aitken, Vaso Totsika, Tania King, Roger J. Stancliffe, Chris Hatton, Gwynnyth Llewellyn, Richard P. Hastings, Anne Kavanagh

**Affiliations:** ^1^ Centre for Disability Research, Faculty of Health & Medicine Lancaster University Lancaster UK; ^2^ College of Nursing and Health Sciences Flinders University Adelaide South Australia Australia; ^3^ Centre of Research Excellence in Disability and Health, Melbourne School of Population and Global Health University of Melbourne Melbourne Victoria Australia; ^4^ Disability and Health Unit, Centre for Health Equity, Melbourne School of Population and Global Health The University of Melbourne Melbourne Victoria Australia; ^5^ Division of Psychiatry University College London London UK; ^6^ Centre for Educational Development, Appraisal and Research University of Warwick Coventry UK; ^7^ Centre for Developmental Psychiatry and Psychology Monash University Clayton Victoria Australia; ^8^ Centre for Disability Research & Policy, Faculty of Medicine and Health University of Sydney Sydney New South Wales Australia; ^9^ Department of Social Care and Social Work Manchester Metropolitan University Manchester UK

**Keywords:** adults, conflict, COVID‐19, disability, stress, trust, wellbeing

## Abstract

Concern has been expressed about the extent to which people with disabilities may be particularly vulnerable to negative impacts of the 2020 COVID‐19 pandemic. However, to date little published research has attempted to characterise or quantify the risks faced by people with/without disabilities in relation to COVID‐19. We sought to compare the impact of the early stages of the COVID‐19 pandemic and associated government responses among working age adults with and without disabilities in the UK on; COVID‐19 outcomes, health and wellbeing, employment and financial security, health behaviours, and conflict and trust. We undertook secondary analysis of data collected in four UK longitudinal surveys; the Millennium Cohort Study, Next Steps, the British Cohort Study and the National Child Development Study. Combining analyses across surveys with random effects meta‐analysis, there was evidence that people with disabilities were significantly more likely to report having had COVID‐19 and had significantly increased levels of stress, less exercise, poorer sleep patterns, more conflict with their partner and others in their local area, and to have less trust in the government. While most outcomes did not differ significantly between participants with and without disability, the findings suggest that in the early days of COVID‐19 a detrimental impact emerges for those with disabilities which is more pronounced among older people with disabilities. Future research is needed to determine the longer‐term impact of the pandemic.

**What is known about this topic**

- Little is known about the impact of the 2020 COVID‐19 pandemic on the well‐being of working age adults with/without disabilities.

**What this paper adds**

- Respondents with disabilities were more likely than their non‐disabled peers to report having all of the three key symptoms of COVID‐19 (fever, cough, loss of taste/smell) in the previous 2 weeks
- They were also more likely to report having increased levels of stress, to sleep less, have more conflict with their partner and others and less trust in the government.
- It will be important for future research to determine the longer‐term impact of the pandemic on such matters as finances, employment, stress, conflict, trust and the health behaviours of people with disabilities.

## 1 INTRODUCTION

People with disabilities are more likely than their non‐disabled peers to be exposed to financial stressors (Heslop & Emerson, 2018; Kavanagh et al., 2016) which are detrimental to health and wellbeing (World Health Organization Regional Office for Europe, 2012). The COVID‐19 pandemic has had a serious impact on the economies of many countries (Committee for the Coordination of Statistical Activities, 2020). Country responses to the pandemic exposed flaws in social systems and highlighted the extent to which different groups are marginalised and disadvantaged in society, with policy responses related to social care focusing largely on older people in congregate care and paying less attention to working age people with disabilities using a wider range of social care supports (Comas‐Herrera, Fernandez, et al., 2020; Comas‐Herrera, Glanz, et al., 2020; Knapp et al., 2021). Considerable concern has been expressed about the extent to which people with disabilities, and those who support them, may be particularly vulnerable to negative impacts of the pandemic (Armitage & Nellums, 2020; Boyle et al., 2020; Flynn et al., 2021; Goggin & Ellis, 2020; Kavanagh et al., 2021; Lund, 2020; Sabatello et al., 2020; Shakespeare et al., 2021; Turk & McDermott, 2020).

However, to date, little published research has attempted to characterise or quantify the risks faced by people with disabilities in relation to COVID‐19. The exceptions have suggested that: COVID‐19 fatality rates are higher among adults with disabilities (Office for Disability National Statistics, 2021); COVID‐19 infection and case fatality rates are higher among people with intellectual or developmental disabilities (Gleason et al., 2021; Henderson et al., 2021; Office for Disability National Statistics, 2021; Office for National Statistics, 2020a; Public Health England, 2020; Turk et al., 2020); in the early stages of the pandemic working age adults with disability were more likely than their peers to be working reduced hours and experience higher levels of financial stress (Emerson et al., 2021); people with mild intellectual disabilities may experience social isolation (Embregts et al., 2020); children with intellectual or developmental disabilities may have reduced access to education and health services (Jeste et al., 2020; Neece et al., 2020); the pandemic may be having a negative impact on the mental health of children and adults with disabilities (Asbury et al., 2020; Flynn et al., 2021; Office for National Statistics, 2020b, 2020c, 2020d); and older people with physical disabilities appear to be at particular risk for emotional distress, poor quality of life, and low wellbeing during the COVID‐19 pandemic (Steptoe & Di Gessa, 2021). Unfortunately, a significant proportion of the published research to date is neither population‐based nor comparative. Although existing research may highlight important pandemic‐related experiences of adults with disabilities, to inform longer term policy it is crucial to generate evidence that may be less affected by sampling biases and that is contextualised by comparison with other groups in society.

The aim of this paper is to redress this omission by comparing the short‐term impact of the COVID‐19 pandemic on outcomes for working age adults with and without disabilities (operationally defined as all adults in the age range 18–64). Specifically, we compared health and wellbeing outcomes related to COVID‐19, employment and financial security, health behaviours, and conflict and trust of adults with and without disabilities participating in four birth cohort studies in the UK.

## 2 METHODS

We undertook secondary analysis of data collected in four UK longitudinal surveys managed by the Centre for Longitudinal Studies at University College London; the Millennium Cohort Study (MCS), Next Steps (NS), the 1970 British Cohort Study (BCS70) and the National Child Development Study (NCDS). Data from these surveys are available through the UK Data Service (http://ukdataservice.ac.uk/). Brief details of the three cohort studies are presented below.

In May 2020 the Centre for Longitudinal Studies and the MRC Unit for Lifelong Health and Ageing carried out an online survey of the participants of five of the UK's national longitudinal cohort studies, including MCS, NS, BCS70 and NCDS. In the UK, stringent ‘lockdown’ rules were enacted in March 2020. During this period, lockdown restrictions affecting all four UK nations included: the compulsory closure of non‐essential businesses and shops; the closure of all schools, pubs, restaurants and cafés; the introduction of working from home wherever possible; restrictions on travel, confining people to their local area; and restrictions in social contact (e.g., only being allowed outside of the home for essential shopping and exercise, ban on in‐home social contact with non‐residents).

The aim of the online survey was to collect information from study participants about the impact of the pandemic on physical and mental health and wellbeing, family and relationships, education, work, and finances during the early stages of COVID‐19 lockdown. The impact questions focussed mainly on how participants' lives had changed from just before the outbreak of the pandemic in March 2020 up until their response to the survey at the height of the lockdown restrictions in May 2020 (Brown et al., 2020). Invitations to participate in the online survey were sent to all cohort members for whom an email address was held and who: (1) had not permanently withdrawn from the study; (2) were not ‘permanently untraced’; and (3) were not known to have died. Response rates, from the immediate prior mainstage wave of data collection, ranged from 20% in NS to 58% in NCDS. In our analyses, we sought to identify indicators of the impact of the early stages of the COVID‐19 pandemic in the UK on lifestyle and wellbeing that either: (1) asked respondents to directly judge the impact of the pandemic by comparing their lifestyle ‘in the three months before the coronavirus outbreak’ with their lifestyle in May 2020; or (2) asked respondents to describe their lifestyle retrospectively ‘in the three months before the coronavirus outbreak’ and, with an identical question format, currently in May 2020 (from which change in lifestyle measures could be derived). Measures included in our analyses are summarised in Table 1. In Table 2, we summarise key aspects of responses to the May 2020 COVID‐19 survey across the three cohort studies.

**TABLE 1:** Outcome variables from may 2020 COVID‐19 questionnaire

Outcomes	Form	Details	Missing data
Health and wellbeing			
Self‐rated health	Pre/post	‘In general, would you say your health is/was … excellent, very good, good, fair, poor’. Recoded as a binary variable; self‐rated health currently poorer than in 3 months preceding pandemic (vs. not)	0.4%
Self‐rated stress	Judgement	‘Since the Coronavirus outbreak please indicate how the following have changed …. The amount of stress I've been feeling … more than before, same—no change, less than before’. Recoded as a binary variable; stress ‘more than before’ (vs. not)	10.6%
Financial status and economic activity			
Financial status	Judgement	‘Overall, how do you feel your *current* financial situation compares to *before* the Coronavirus outbreak? …. I'm much worse off, I'm a little worse off, I'm about the same, I'm a little better off, I'm much better off’. Recoded as a binary variable; finance ‘worse off’ (vs. not)	5.8%
Economic activity	Pre/post	‘Which of these best describes what you were doing just *before* the Coronavirus outbreak/*currently*? If you were doing more than one activity, please choose the activity that you spent most time doing …. employed, self‐employed, in unpaid/voluntary work, apprenticeship, unemployed, permanently sick or disabled, looking after home or family, in education at school/college/university, retired, doing something else’. Recoded as a binary variable; economic activity ‘lost employment (i.e., moved from employed or self‐employed to non‐employed category’ (vs. not)	0.9%
Health behaviours			
Cigarettes smoked	Pre/post	‘In the month *before* the Coronavirus outbreak/since the outbreak, how many cigarettes a day did/do you usually smoke?’ Recoded as a binary variable; currently smoking more cigarettes a day than previously (vs. not)	7.5%
Vaping	Judgement	‘Since the start of the Coronavirus outbreak, has the amount you have been using an electronic cigarette or vaping device changed? Yes—I have used an electronic cigarette or vaping device more often, Yes—I have used an electronic cigarette or vaping device less often, No Recoded as a binary variable; currently vaping more than previously (vs. not)	8.0%
Alcohol use	Pre/post	‘In the month *before/since the start* of the Coronavirus outbreak, how many standard alcoholic drinks do you have on a typical day when you were drinking? 1–2, 3–4, 5–6, 7–9, 10+’ Recoded as a binary variable; currently drinking more than previously (vs. not)	7.4%
Exercise	Pre/post	‘In the month *before/since the start* of the Coronavirus outbreak, on how many days in a typical week did you do 30 min or more of exercise where you are working hard enough to raise your heart rate and break into a sweat?’ Recoded as a binary variable; exercising for fewer days than previously (vs. not)	9.7%
Fruit/veg consumption	Pre/post	‘In the month *before/since the start* of the Coronavirus outbreak, how many portions of fresh fruit and vegetables did you eat in a typical day? [A portion of fruit could be a whole piece of fruit, like an apple or banana or 80 g of fruit (like in a fruit salad). A portion of vegetables is 3 heaped tablespoons of cooked vegetables or beans/pulses or a handful of cherry tomatoes or a small bowl of salad. It does not include potatoes. Juice/smoothies can count as 1 portion per day.]’ Recoded as a binary variable; eating fewer portions of fruit/veg than previously (vs. not)	12.0%
Sleep	Pre/post	‘In the month *before/since the start* of the Coronavirus outbreak, how many hours did you sleep each night on average?’ Recoded as a binary variable; sleeping for fewer hours than previously (vs. not)	8.6%
Conflict and trust			
Relationship conflict	Judgement	‘Since the Coronavirus outbreak began, has the amount you have argued with your partner changed? My partner and I have argued more often, No change—same as before, My partner and I have argued less often.’ Recoded as a binary variable; conflict ‘more than before’ (vs. not). Note: missing data calculated for respondents in a cohabiting or non‐cohabiting relationship.	0.5%
Conflict with other people	Judgement	‘Since the Coronavirus outbreak please indicate how the following have changed …. The amount of conflict I have had with people around me … more than before, same—no change, less than before’. Recoded as a binary variable; conflict ‘more than before’ (vs. not)	10.9%
Trust in government	Judgement	‘Since the Coronavirus outbreak please indicate how the following have changed …. The amount of trust I have in the Government … more than before, same—no change, less than before’. Recoded as a binary variable; trust ‘less than before’ (vs. not)	11.1%
Trust in people in local area	Judgement	‘Since the Coronavirus outbreak please indicate how the following have changed …. The amount of trust I have in people in my local area … more than before, same—no change, less than before’. Recoded as a binary variable; trust ‘less than before’ (vs. not)	10.8%

*Note*: *Pre/post*—respondents were asked to retrospectively estimate lifestyle in the 3 months prior to pandemic and, separately, in May 2020. *Judgement*—respondents were asked to estimate change in lifestyle comparing the 3 months prior to pandemic with May 2020.

**TABLE 2:** Characteristics of respondents to the may 2020 COVID‐19 questionnaire for the four cohort studies

	Millennium Cohort Study	Next Steps	BCS70	National Child Development Study
Response rate	26.6%	20.3%	40.4%	57.9%
Sample size (unweighted, total)	2629	1858	4223	5178
Sample size (unweighted, with valid disability information)	2575	1706	3656	4837
Age (in years) in May 2020	19	30	50	62
Age (in years) when disability status was determined	17	25	46	55
Weighted % female	50.6%	56.6%	48.7%	51.0%
Weighted % with disability	9.7%	11.9%	21.1%	20.2%
Living situation				
With disability: living with partner	6.5%	44.8%	57.3%	78.6%
No disability: living with partner	6.9%	67.8%	72.8%	83.3%
With disability: living with parent(s)	85.3%	36.4%	5.8%	2.4%
No disability: living with parent(s)	87.7%	17.8%	3.8%	2.7%

### 2.1 The cohort studies

#### 2.1.1 Millennium cohort study

Millennium cohort study is the latest of the UK's birth cohort studies, designed to follow a sample of children, born around 2000, through their life. Full details of the design of MCS are available in a series of reports and technical papers (Fitzsimons et al., 2020).

Participant families were randomly selected from Child Benefit Records, a universal welfare benefit available at the time to all UK children. Sampling was geographically clustered in 398 randomly selected electoral wards in the UK and stratified to over‐sample children from ethnic minority groups, disadvantaged communities and children in Scotland, Wales and Northern Ireland (Plewis, 2007). The first survey took place when children were 9 months old and included a total of 18,551 families. Data have been made available to date from children followed up at ages 3, 5, 7, 11, 14 and 17 years. For each family, information was collected on the target child falling within the designated birth date window. For multiple births (e.g., twins, triplets) information was collected on each child. At the latest available wave of data collection (at age 17) information was collected from the cohort child for 10,834 children (58% retention from Wave 1).

#### 2.1.2 Next Steps

Next Steps (formerly known as the *Longitudinal Study of Young People in England*) was an annual panel study that followed a cohort of children from early adolescence into adulthood. NS is currently managed by the Centre for Longitudinal Studies at University College London and is funded by the Economic and Social Research Council. Prior to 2013, NS was managed and funded by the Department for Education. *NS* data files and documentation were obtained from the UK Data Service. Full details of the method and design of *NS* are available in a series of user guides (Calderwood, 2017).

Fieldwork commenced in 2004 when the sampled children were aged 13–14 years. The initial sample was drawn from a sampling frame based on children attending state funded schools, independent schools and pupil referral units in England, who in February 2004 were in Year 9 (13–14 years of age) and were born between 1 September 1989 and 31 August 1990. Schools in deprived areas and students from minority ethnic groups were oversampled. 73% of selected schools participated in the initial sample leading to an issued sample of approximately 21,000 young people and an attained sample of 15,770 children (75% response rate). This cohort was followed‐up every year until age 19–20 and then at age 25. At the last wave of data collection (age 25) information was collected by a combination of online, telephone, and computer assisted personal interview from 7707 cohort members (49% retention from Wave 1).

#### 2.1.3 1970 British Cohort Study

BCS70 is one of the UK's birth cohort studies, designed to follow a sample of children born in a particular week in 1970 through their life. Full details of the design of BCS70 are available in cohort profiles (Brown, 2014; Elliott & Shepherd, 2006) and in a series of technical reports and supporting documentation (e.g., interview questionnaires) that are available for download from the UK Data Service (https://www.ukdataservice.ac.uk/).

BCS70 has followed up over 17,000 children born during 1 week in the UK in 1970. In the first wave of data collection (soon after birth) information was collected from midwives on 17,198 infants (the cohort members). Since then, information has been collected on various aspects of the lives of cohort members at age 5, 10, 16, 26, 30, 34, 38, 42, and 46 years (Brown & Peters, 2019). At the latest available wave of data collection (2016) information was collected by computer assisted personal interview at age 46 from 8581 cohort members (50% retention from Wave 1).

### 2.2 National Child Development Study

National Child Development Study is a UK birth cohort study designed to follow a sample of children born in a particular week in 1958 through their life. Full details of the design of NCDS are available in a cohort profile (Power & Elliott, 2006) and in a series of technical reports and supporting that are available for download from the UK Data Service (e.g., Brown & Hancock, 2015). NCDS has followed up just over 17,000 children who were originally part of the Perinatal Mortality Survey. Since birth, information has been collected on various aspects of the lives of cohort members at age 7, 11, 16, 23, 33, 42, 44, 45, 50 and 55 years. At the latest available wave of data collection (2013) information was collected by computer assisted personal interview at age 55 from 9137 cohort members (54% retention from birth).

### 2.3 Disability

Disability data were not collected in the COVID May 2020 COVID‐19 online surveys. As a result and following an existing precedent (Steptoe & Di Gessa, 2021), we identified disability status using data collected from the cohort member at the latest preceding available wave of the main survey (Understanding Society 2017–2019, MCS 2018–19, NS 2015–16, BCS70 2016–18, NCDS 2013–14). We defined cohort members as having a disability if they reported that they: (1) had ‘physical or mental health conditions or illnesses lasting or expected to last 12 months or more’; (2) that this reduced their ‘ability to carry out day‐to‐day activities’; and (3) that this limitation had lasted for at least 6 months. The prevalence of disability increased with age in the samples responding to the COVID‐19 surveys from 9.7% (95% CI 8.7–10.9) in MCS (at age 17), 11.9% (95% CI 10.4–13.5) in NS (at age 25), 21.1% (95% CI 19.8–22.5) in BCS70 (at age 46) and 20.2% (95% CI 19.1–21.3) in NCDS (at age 55).

### 2.4 Covariates

No data were missing from the analytical sample on basic demographic variables (gender) which were included in models to control for potential confounding. We also adjusted for family socio‐economic position during the cohort member's childhood. For each cohort, we derived an indicator of: (1) likely income poverty; and where possible (2) local area deprivation.

- For MCS, measures were taken at age 9 months, age 3, and age 5. The measure of likely income poverty was whether household equivalised income was below 60% of the national median, one of the ‘headline’ measures of poverty used by the UK government (Department of Work and Pensions, 2003). The measure of local area deprivation was whether the family was living in an area in the highest national quintile of deprivation as measured by the relevant national Indices of Deprivation (Department for Communities and Local Government, 2015).
- For NS, measures were taken at age 13/14 and 15/16 years. The measure of likely income poverty was whether the child was eligible for Free School Meals, a commonly used measure of poverty in UK education research (Kounali et al., 2008). The measure of local area deprivation was whether the family was living in an area in the highest national quintile of deprivation as measured by the English Income Deprivation Affecting Children Index (Department for Communities and Local Government, 2015).
- For BCS70, measures were taken at age 5 and 10 years. The measure of likely income poverty was whether the child was living in a family in which no adult was in paid employment. The measure of local area deprivation was whether at age 5, the family was recorded as living in a ‘poor’ area and at age 10 whether they were living in a ‘Council estate’ (estates of local government provided social housing).
- For NCDS, likely income poverty was defined whether the child's accommodation lacked sole use of any of three basic amenities (bath, hot water, kitchen) at any of the three ages at which the data were collected (age 7, 11 and 16).

For each of the two indicators we derived a simple count measure of low family socio‐economic position (not exposed, exposed once, exposed more than once).

### 2.5 Ethical review

Ethical review procedures for the cohort surveys and the online COIVID‐19 survey are outlined in a series of reports available from the Centre for Longitudinal Studies 2020 (Brown, 2014; Brown & Hancock, 2015; Brown et al., 2020; Calderwood, 2017; Elliott & Shepherd, 2006; Fitzsimons et al., 2020; Power & Elliott, 2006).

### 2.6 Approach to analysis

The analytical sample comprised all 12,774 respondents aged 18–64 to the COVID‐19 survey for May 2020 for whom valid disability data were available from previous waves of the longitudinal surveys. Missing data on outcome variables are presented in Table 1. Complete case analyses were undertaken in Stata 16 using the sample weights released with the COVID‐19 data to account for known biases in recruitment and retention. These weights were recalibrated to ensure that the weighted sample size was identical to the unweighted sample size. For MCS and NS, we also used the svyset/svy routines to take account of the clustered sample design (BCS70 and NCDS sampling did not involve clustering). Unless stated, Poisson regression was used to estimate adjusted prevalence rate ratios (APRRs) with 95% confidence intervals (Knol et al., 2012).

First, for each cohort, we estimated the percentage of people with/without disability experiencing each outcome (with 95% confidence intervals). In addition, we estimated APRRs for respondents with disabilities being exposed to each outcome (respondents without disabilities being the reference group). In Model 1 we adjusted for between‐group differences in gender (male, female). In Model 2, to address the possible confounding effects of family socio‐economic position on outcomes, we also adjusted for childhood socio‐economic position. Second, we used random effects meta‐analysis in Stata 16.1 to combine results across cohorts. We report summary statistics for effect sizes (with 95% confidence intervals) and *I* ^2^, which represents the proportion of total variation in study estimates that is due to heterogeneity (Higgins et al., 2003). However, given the bias in *I* ^2^ when the number of studies is small (as in the present case), *I* ^2^ statistics should be treated with some caution (von Hippel, 2015). Given the limitations associated with a sole reliance on null hypothesis significance testing to identify results that may be of social or policy relevance (Wasserstein & Lazar, 2016), we also draw attention in the text to results with notable effect sizes that are not statistically significant (*p* < 0.05), but which may warrant further investigation given their potential importance.

## 3 RESULTS

Prevalence rates for outcomes and APRRs for each cohort are presented in Table 3. Pooled results across the three cohorts are presented in Table 4.

**TABLE 3:** Outcomes disaggregated by cohort

Outcome	Millennium Cohort Study (19)^a^				Next Steps (30)^a^				BCS70 (50)^a^				National Child Development Study (62)^a^			
	PWD	Others	APRR1	APRR2	PWD	Others	APRR1	APRR2	PWD	Others	APRR1	APRR2	PWD	Others	APRR1	APRR2
COVID‐19																
Has had COVID	7.7% (4.2–13.7)	5.0% (3.9–6.4)	1.48 (0.77–2.82)	1.46 (0.77–2.80)	8.1% (4.5–14.3)	10.2% (8.0–13.0)	0.82 (0.44–1.52)	0.81 (0.43–1.53)	12.7% (10.2–15.7)	9.1% (8.1–10.1)	1.39 (0.81–2.39)	1.00 (0.69–1.46)	8.7% (6.9–10.9)	4.9% (4.3–5.6)	1.76 (1.11–2.80)*	1.43 (0.99–2.07)
Potential key COVID symptom in last 2 weeks	19.5% (13.9–26.7)	22.3% (19.3–25.6)	0.87 (0.61–1.24)	0.88 (0.62–1.24)	16.1% (9.9–25.3)	14.9% (11.9–18.6)	1.07 (0.65–1.76)	1.05 (0.64–1.73)	40.7% (36.7–44.8)	18.1% (16.7–19.4)	2.27 (1.80–2.88)***	2.03 (1.72–2.41)***	28.7% (25.6–31.9)	18.8% (17.7–20.1)	1.52 (1.22–1.89)***	1.48 (1.20–1.82)***
Fever	3.6% (1.6–7.7)	2.5% (1.8–3.6)	1.36 (0.58–3.16)	1.28 (0.57–2.87)	3.8% (1.7–8.2)	4.1% (2.4–6.6)	0.89 (0.35–2.23)	0.92 (0.35–2.38)	12.7% (10.2–15.7)	3.2% (2.7–3.9))	3.91 (1.85–8.28)***	2.61 (1.39–4.92)**	3.4% (2.3–4.9)	2.1% (1.7–2.6)	1.52 (0.89–2.59)	1.82 (1.06–3.12)*
Cough	17.9% (12.5–25.0)	20.0% (17.0–23.3)	0.90 (0.61–1.31)	0.90 (0.62–1.31)	14.5% (8.5–23.6)	11.3% (8.9–14.4)	1.28 (0.74–2.22)	1.25 (0.73–2.15)	36.0% (32.2–40.1)	15.7% (14.4–17.0)	2.30 (1.77–3.00)***	2.03 (1.69–2.42)***	25.9% (23.0–29.1)	16.4% (15.3–17.7)	1.59 (1.25–2.01)***	1.48 (1.18–1.86)**
Loss of taste or smell	1.9% (0.9–4.0)	2.6% (1.7–4.0)	0.72 (0.31–1.64)	0.68 (0.30–1.58)	2.3% (0.7–7.2)	3.2% (1.8–5.6)	0.69 (0.18–2.51)	0.78 (0.21–2.84)	7.7% (5.7–10.2)	4.1% (3.4–4.8)	1.87 (0.86–4.08)	1.86 (1.12–3.10)*	4.9% (3.6–6.6)	2.9% (2.4–3.4)	1.63 (0.90–2.96)	1.99 (1.14–3.45)*
Tested for COVID	4.0% (1.7–9.2)	2.1% (1.3–3.6)	1.92 (0.68–4.43)	1.95 (0.70–5.41)	6.8% (3.0–14.9)	3.7% (2.2–6.2)	1.81 (0.70–4.65)	1.99 (0.76–5.16)	2.9% (1.8–4.7)	3.7% (3.1–4.4)	0.78 (0.36–1.66)	0.73 (0.44–1.24)	4.5% (3.2–6.1)	2.2% (1.8–2.7)	1.99 (0.76–5.18)	1.56 (0.81–2.99)
Tested for COVID if symptomatic in last 2 weeks	7.6% (1.9–26.3)	4.1% (1.9–8.9)	1.63 (0.25–10.72)	1.54 (0.30–7.83)	14.8% (4.7–37.8)	13.3% (6.6–24.9)	1.22 (0.35–4.27)	2.02 (0.61–6.65)	4.5% (2.9–7.0)	9.5% (8.2–11.0)	0.32 (0.15–0.69)**	0.55 (0.24–1.22)	12.3% (8.5–17.5)	2.7% (1.7–4.1)	4.63 (1.44–14.89)*	1.94 (0.66–5.76)
Admitted to hospital for COVID	0.0% (0.0–5.7)	0.5% (0.2–1.5)	n/a	n/a	2.1% (0.5–8.4)	2.1% (0.6–7.0)	0.82 (0.12–5.60)	0.95 (0.12–7.38)	0.4% (0.1–2.6)	0.9% (0.5–1.8)	0.37 (0.06–2.21)	0.42 (0.12–1.52)	1.2% 0.4–3.6)	0.7% (0.3–1.5)	1.75 (0.52–5.84)	1.86 (0.55–6.22)
Self‐rated health and stress																
Self‐rated health poorer	2.6% (1.2–5.6)	2.6% (1.5–4.4)	1.00 (0.41–2.43)	1.03 (0.43–2.47)	4.8% (2.0–11.1)	1.0% (0.6–1.9)	4.70 (1.66–13.35)**	4.59 (1.75–12.07)**	1.8% (1.0–3.4)	1.8% (1.3–2.3)	0.74 (0.26–2.16)	0.80 (0.37–1.73)	3.2% (2.2–4.7)	1.6% (1.3–2.1)	2.08 (1.09–3.97)*	2.13 (1.07–4.22)*
Stress increased	61.5% (53.7–68.7)	35.2% (32.1–38.4)	1.59 (1.34–1.87)***	1.57 (1.34–1.85)***	61.5% (48.7–72.9)	42.8% (38.6–47.1)	1.42 (1.12–1.80)**	1.43 (1.13–1.81)**	49.3% (45.1–53.6)	35.0% (33.3–36.8)	1.37 (1.16–1.62)***	1.29 (1.15–1.45)***	40.0% (36.4–43.5)	31.1% (29.6–32.6)	1.19 (1.01–1.41)*	1.30 (1.13–1.50)***
Health behaviours																
Smoking more^b^	5.9% (3.6–9.6)	5.8% (4.7–7.2)	0.71 (0.42–1.18)	0.79 (0.48–1.30)	33.3% (12.5–63.7)	20.5% (12.4–32.0)	1.49 (0.58–3.81)	1.41 (0.57–3.47)	18.2% (11.0–28.5)	13.3% (10.1–17.3)	1.42 (0.63–3.19)	1.29 (0.76–2.19)	5.2% (1.8–14.0)	9.6% (6.8–13.5)	0.53 (0.15–1.86)	0.23 (0.07–0.81)*
Vaping more^b^	72.4% (46.0–89.0)	43.9% (30.8–58.0)	1.65 (1.04–2.62)*	1.44 (0.91–2.04)	44.7% (15.7–77.8)	49.3% (32.8–66.0)	0.91 (0.37–2.22)	1.34 (0.53–3.41)	20.4% (11.9–32.8)	25.2% (19.7–31.6)	0.82 (0.36–1.87)	0.86 (0.48–1.54)	40.1% (27.2–54.5)	28.5% (22.0–36.0)	1.49 (0.81–2.71)	1.47 (0.81–2.67)
Drinking more	32.5% (25.1–40.9)	42.2% (39.1–45.4)	0.67 (0.49–0.91)*	0.71 (0.53–0.94)*	32.8% (20.0–48.9)	19.8% (16.2–24.0)	1.65 (0.99–2.75)	1.59 (1.00–2.53)*	16.8% (13.3–21.0)	9.7% (8.60–10.9)	1.65 (1.01–2.71)**	1.53 (1.09–2.14)*	7.6% (5.7–10.1)	9.5% (8.5–10.5)	0.85 (0.61–1.18)	0.87 (0.63–1.22)
Exercising less	31.7% (21.7–43.7)	29.2% (26.3–32.3)	1.13 (0.79–1.62)	1.13 (0.86–1.50)	30.2% (19.1–44.3)	29.6% (25.9–33.7)	1.03 (0.67–1.59)	1.02 (0.66–1.57)	24.8% (21.2–28.8)	20.2% (18.8–21.7)	1.19 (0.88–1.62)	1.19 (0.96–1.47)	20.2% (17.5–23.3)	17.6% (16.4–18.8)	1.15 (0.83–1.55)	0.93 (0.74–1.17)
Less fruit and veg	8.1% (4.8–13.3)	15.1% (13.1–17.3)	0.57 (0.31–0.90)*	0.57 (0.33–0.97)*	22.7% (14.7–30.2)	14.7% (12.3–17.6)	1.54 (1.01–2.35)*	1.57 (1.03–2.38)*	19.1% (15.9–22.8)	14.5% (13.2–15.8)	1.30 (0.92–1.84)	1.19 (0.94–1.51)	17.0% (14.4–20.0)	8.1% (7.4–9.1)	1.94 (1.37–2.75)***	1.98 (1.41–2.77)***
Sleeping less	22.5% (16.4–30.1)	21.5% (18.5–24.9)	1.09 (0.74–1.47)	1.11 (0.79–1.55)	40.5% (28.2–54.2)	19.6% (16.8–22.8)	2.05 (1.41–3.00)***	2.09 (1.44–3.03)***	20.9% (17.6–24.7)	18.6% (17.2–20.1)	1.14 (0.87–1.49)	1.20 (1.00–1.45)*	25.3% (22.2–28.5)	14.4% (13.3–15.5)	1.61 (1.21–2.14)**	1.47 (1.13–1.93)**
Conflict and trust																
More conflict with partner	36.8% (25.4–50.1)	31.9% (27.5–36.6)	1.12 (0.78–1.59)	1.17 (0.83–1.65)	23.2% (13.3–37.4)	18.5% (14.9–22.8)	1.22 (0.70–2.11)	1.19 (0.69–2.06)	14.7% (11.6–18.3)	9.0% (8.0–10.2)	1.56 (1.02–2.38)*	1.57 (1.19–2.08)**	16.5% (13.8–19.7)	7.3% (6.5–8.2)	2.18 (1.39–3.43)**	1.88 (1.28–2.76)**
More conflict with others	25.5% (18.6–33.8)	20.0% (17.7–22.6)	1.22 (0.88–1.70)	1.23 (0.89–1.70)	21.3% (12.6–33.6)	8.9% (6.8–11.5)	2.37 (1.35–4.17)**	2.39 (1.37–4.18)**	9.0% (6.8–11.8)	7.6% (6.7–8.6)	1.15 (0.74–1.79)	1.20 (0.86–1.68)	9.4% (7.5–11.7)	3.5% (3.0–4.1)	2.57 (1.52–4.34)***	2.16 (1.35–3.44)**
Less trust in government	33.7% (25.6–43.0)	27.3% (23.6–31.3)	1.23 (0.93–1.64)	1.22 (0.91–1.64)	47.1% (34.4–60.1)	28.7% (25.2–32.5)	1.65 (1.20–2.27)**	1.65 (1.21–2.25)**	30.8% (27.0–34.8)	21.2% (19.8–22.7)	1.44 (1.11–1.87)***	1.27 (1.05–1.53)*	29.7% (26.5–33.1)	22.6% (21.3–24.0)	1.33 (1.04–1.68)*	1.27 (1.02–1.57)*
Less trust in others	13.2% (8.5–19.9)	16.5% (13.9–19.4)	0.78 (0.50–1.23)	0.77 (0.48–1.22)	20.1% (11.7–32.4)	12.7% (9.9–16.2)	1.58 (0.90–2.79)	1.60 (0.91–2.80)	14.8% (12.0–18.1)	7.0% (6.2–8.0)	2.11 (1.36–3.28)***	1.62 (1.18–2.24)**	7.1% (5.4–9.1)	5.4% (4.7–6.2)	1.24 (0.59–2.58)	1.34 (0.65–2.81)
Employment and financial situation																
Lost employment	15.5% (7.9–28.2)	17.2% (13.0–22.4)	1.04 (0.54–1.91)	0.97 (0.52–1.81)	4.2% (1.8–9.2)	8.1% (5.9–11.2)	0.49 (0.21–1.14)	0.49 (0.22–1.13)	13.8% (10.7–17.5)	12.4% (11.2–13.7)	1.13 (0.73–1.75)	0.97 (0.73–1.29)	16.1% (12.6–20.4)	18.4% (16.8–20.0)	0.92 (0.65–1.29)	0.91 (0.65–1.28)
Financially worse off	29.8% (22.4–38.4)	27.5% (24.5–30.7)	1.08 (0.82–1.42)	1.08 (0.82–1.43)	19.5% (9.4–36.1)	11.6% (8.7–15.2)	1.68 (0.80–3.50)	1.11 (0.78–1.60)	37.7% (33.7–41.8)	35.0% (33.3–36.7)	1.07 (0.66–1.44)	1.11 (0.96–1.28)	28.8% (25.7–32.1)	32.0% (30.6–33.5)	0.92 (0.74–1.16)	0.89 (0.75–1.10)

Abbreviation: PWD, people with disabilities.

^a^ Cohort age at time of COVID‐19 survey.

^b^ Analyses restricted to respondents who smoked/vaped pre‐pandemic.

* *p* < 0.05

** *p* < 0.01

*** *p* < 0.001.

**TABLE 4:** Adjusted prevalence rate ratios (APPR) combined across cohorts comparing outcomes between people with and without disability

	APRR1	*I* ^2^	APRR2	*I* ^2^
COVID‐19				
Has had COVID	1.38 (1.06–1.79)*	7.4	1.16 (0.90–1.51)	18.5
Key COVID symptom in last 2 weeks	1.39 (0.91–2.10)	95.1	1.33 (0.91–1.93)	87.3
Fever	1.75 (0.92–3.29)	71.5	2.01 (1.45–2.80)***	0.0
Cough	1.48 (0.98–2.23)	86.5	1.40 (0.99–1.99)	82.7
Loss of taste or smell	1.28 (0.79–2.10)	41.4	1.36 (0.80–2.32)	54.5
Tested for COVID	1.36 (0.78–2.39)	43.8	1.30 (0.75–2.27)	54.2
Tested for COVID if key COVID symptom in last 2 weeks	1.11 (0.33–3.74)	76.2	1.19 (0.52–2.72)	60.7
Admitted to hospital for COVID	0.82 (0.29–2.34)	38.8	0.89 (0.30–2.61)	39.0
Self‐rated health and stress				
Self‐rated health poorer	1.58 (0.74–3.39)	71.0	1.65 (0.79–3.42)	69.3
Stress increased	1.38 (1.23–1.55)***	49.1	1.38 (1.24–1.51)***	36.1
Health behaviours				
Smoking more^a^	0.99 (0.62–1.58)	39.1	0.88 (0.50–1.55)	60.4
Vaping more^a^	1.23 (0.85–1.79)	34.4	1.26 (0.94–1.70)	0.0
Drinking more	1.09 (0.70–1.73)	84.3	1.08 (0.73–1.62)	80.9
Exercising less	1.16 (1.00–1.34)*	0.0	1.07 (0.93–1.23)	10.1
Less fruit and veg	1.26 (0.78–2.03)	85.2	1.23 (0.74–2.03)	86.8
Sleeping less	1.40 (1.06–1.86)*	73.4	1.39 (1.09–1.79)**	67.7
Conflict and trust				
More conflict with partner	1.46 (1.05–1.73)*	32.9	1.40 (1.11–1.93)**	49.1
More conflict with others	1.63 (1.08–2.46)*	74.7	1.58 (1.12–2.23)**	65.1
Less trust in government	1.40 (1.25–1.58)***	0.0	1.31 (1.16–1.48)***	0.0
Less trust in others	1.36 (0.86–2.18)	72.5	1.28 (0.87–1.87)	58.6
Employment and financial situation				
Lost employment	0.99 (0.80–1.21)	0.0	0.90 (0.77–1.06)	0.0
Financially worse off	1.04 (0.93–1.16)	0.0	1.04 (0.92–1.17)	18.5

*Note*: APRR1 adjusted for gender; APRR2 adjusted for gender and childhood socio‐economic position. *I* ^2^ the percentage of total variation in study estimates that is due to heterogeneity.

^a^ Analyses restricted to respondents who smoked/vaped pre‐pandemic.

* *p* < 0.05

** *p* < 0.01

*** *p* < 0.001.

In the combined results (Table 4), Participants with disabilities were significantly more likely to report having had COVID‐19 (APRR1 = 1.38 [1.06–1.79], *p* < 0.05). In addition, there were moderate (but statistically non‐significant) effect sizes to indicate that people with disabilities were more likely than their non‐disabled peers to report having all three of the key symptoms identified by the NHS of a possible COVID‐19 infection in the previous 2 weeks; fever (APRR1 = 1.75 [0.91–2.10]), cough (APRR1 = 1.48 [0.98–2.23]) and loss of taste/smell (APRR1 = 1.28 [0.79–2.10]).

With regard to social and psychological outcomes, relative to their pre‐pandemic status, people with disabilities were statistically significantly more likely than their non‐disabled peers to report having increased levels of self‐rated stress (APRR1 1.38 [1.23–1.55], *p* < 0.001), to be sleeping less (APRR1 1.40 [1.06–1.86], *p* < 0.05), exercising less (APRR1 1.16 [1.00–1.34], *p* < 0.05), to be having more conflict with their partner (APRR1 1.46 [1.05–1.73], *p* < 0.05) and others in their local area (APRR2 1.63 [1.08–2.46], *p* < 0.05) and to have less trust in the government (APRR1 1.40 [1.25–1.58], *p* < 0.001). There was no statistical evidence of a difference between people with and without disability for other health behaviours and self‐rated health. Further adjusting for the potentially confounding effects of childhood socio‐economic position had only minor effects on reported effect sizes.

Inspection of results for each specific cohort (Table 3) indicates that increased rates of key symptoms for people with disabilities were particularly pronounced among the age 50 and age 62 cohorts. The age 30 and age 62 cohorts reported marked and significant deterioration in their self‐rated health following the onset of the COVID‐19 pandemic. Finally, the age 30 and age 50 cohorts reported increased rates of alcohol consumption following the onset of the COVID‐19 pandemic. Again, further adjusting for the potentially confounding effects of childhood socio‐economic position had only minor effects on reported effect sizes.

## 4 DISCUSSION

While most outcomes did not differ significantly between participants with and without disability, combined analyses (Table 4) indicated that they were significantly more likely to report having COVID‐19 and provided some indication (though not statistically significant) that they were more likely than their non‐disabled peers to report having at least one of the three key symptoms of COVID‐19 in the previous 2 weeks. With regard to social and psychological outcomes, people with disabilities were significantly more likely than their non‐disabled peers to report having increased levels of stress, reduced levels of exercise, poorer sleep patterns, more conflict with their partner and others in their neighbourhood and less trust in the government. Inspection of results for each specific cohort (Table 3) indicated that: (1) increased rates of key symptoms were particularly pronounced among the age 50 and 62 cohorts; (2) the age 30 and 62 cohorts reported marked and significant deterioration in their self‐rated health following the onset of the COVID‐19 pandemic; and (4) the age 30 and/or age 50 cohorts reported increased rates of alcohol consumption following the onset of the COVID‐19 pandemic.

The primary contribution of these analyses is to provide population‐based evidence from four national surveys on wellbeing of people with disabilities at different ages during the early stages of the COVID‐19 pandemic in the UK (during the first lockdown). While most outcomes did not differ significantly between participants with and without disability, the results are consistent with the both concerns that have been expressed about and the limited empirical evidence currently available on the pandemic having a detrimental impact on the wellbeing of people with disabilities (Armitage & Nellums, 2020; Boyle et al., 2020; Emerson et al., 2021; Goggin & Ellis, 2020; Kavanagh et al., 2021; Lund, 2020; Office for National Statistics, 2020b, 2020c, 2020d; Sabatello et al., 2020; Steptoe & Di Gessa, 2021; Turk & McDermott, 2020).

The results add to the existing evidence base in two important ways. First, people with disabilities are at higher risk than their non‐disabled peers of reporting more conflict with their partner (and to an extent others) and lower levels of trust in government. The latter association is likely to reflect the failure of the UK government to adequately address the situation of people with disabilities in the early stages of the pandemic (Comas‐Herrera, Glanz, et al., 2020; Kavanagh et al., 2021). These are potentially important results given that exposure to conflict and low levels of trust have both been identified as potential determinants of poorer health and may potentially have an impact on vaccine hesitancy (Kawachi & Berkman, 2014; World Health Organization, 2014).

Second, the differences between results for the four cohorts raise questions about the possible role of age or living circumstances that change across the life course in moderating the impact of the pandemic on the wellbeing of adults with disabilities. For example, while older people with disabilities were much more likely to report the presence of key symptoms in the previous 2 weeks, those who were symptomatic were less likely to be tested. It will be important for future research to: (1) determine whether similar patterns are evident in other samples; and (2) to investigate potential mechanisms (e.g., the intersection of age and disability on institutional discrimination in health care systems, the buffering role of parental support for people with disabilities at younger ages) that may underlie such patterns. It may be particularly important to further investigate the longer‐term impact of the pandemic on adults aged in their 30s given that the psychological and social impact that can be seen at this age may lay the foundation for poorer physical and mental health in the future.

The importance of these results needs to be considered in light of the limitations of the present study. First, response rates to the online COVID‐19 survey were low (20%–25% at younger ages, 40%–58% at older ages), although not untypical of nationally representative online surveys. This does, however, introduce potential selection bias if the associations between disability and outcomes were different among respondents and non‐respondents. While our use of attrition weights may have reduced bias to an extent, we recommend caution in generalising our findings given the poor response rate. Second, while internet access in the UK is generally very high, the use of an online response format is likely to have led to reduced response rates among participants with disabilities associated with reduced cognitive capacity. Third, there was a significant time lag (2–7 years) between the determination of disability status from data collected in the latest available main wave of the survey and participation in the COVID‐19 survey (for another example of using this approach, see Steptoe & Di Gessa, 2021). Given the dynamic nature of some disabilities (Office for National Statistics, 2012), a proportion of people identified as having a disability will no longer have had a disability by the time of the COVID‐19 survey and vice versa. These putative classification changes may have introduced bias in an unknown direction. Fourth, the dependent variables from the COVID‐19 questionnaire were either retrospective pre‐/post‐COVID‐19 items, or judgements of change compared to before the pandemic. While these variables capture pandemic‐related change, they are not as compelling as prospective longitudinal data. Finally, the use of different cohorts may be considered a limitation due to differences in the methods used, different sampling frames (UK vs. England) and the different ages of participants. It could, however, also be considered a strength of the study through increasing the diversity of the sample and most importantly the power of the study. The study's other main strength lies in its use of longitudinal data to determine disability status prior to the onset of the COVID‐19 pandemic. This reduces the possibility of reverse causation and the likelihood of differential and/or dependent misclassification where effect estimates may be biased away from the null in an unknown direction (VanderWeele & Hernán, 2012).

It is important to keep in mind that the present paper addresses the health and wellbeing of people with disabilities during the very early stages of the COVID‐19 pandemic in the UK. It will be critically important for future research to determine the longer‐term impact of the pandemic and responses to the pandemic (including access to vaccination) on such matters as finances, employment, stress, conflict, trust and health behaviours.

## AUTHOR CONTRIBUTIONS

Eric Emerson undertook all analyses and drafted the paper. All other authors contributed to: (1) framing the aims and methods used in the analyses; (2) agreeing the specific analytic techniques to be employed; (3) the drafting on the initial paper and subsequent revisions. All authors read and agreed with the content of the final submitted manuscript.

## FUNDING INFORMATION

The research was supported by Australian National Health and Medical Research Council grant APP1116385.

## CONFLICT OF INTEREST

None declared.

## Data Availability

The data that support the findings of this study are available from the UK Data Service. Restrictions apply to the availability of these data, which were used under license for this study. Data are available at https://ukdataservice.ac.uk/ with the permission of the UK Data Service.
